# Clinical Characteristics of Mechanically Ventilated Patients Treated in Non-ICU Settings in a Rural Area of Japan

**DOI:** 10.7759/cureus.20931

**Published:** 2022-01-04

**Authors:** Yoshiaki Iwashita, Shinnnosuke Morimoto, Sukenari Koyabu, Kazuo Maruyama, Hiroshi Imai

**Affiliations:** 1 Department of Emergency and Critical Care Medicine, Faculty of Medicine, Shimane University, Izumo, JPN; 2 Department of Internal Medicine, Kinan Hospital, Mihamacho, JPN; 3 Department of Internal Medicine, Owase General Hospital, Owase, JPN; 4 Department of Anesthesiology and Critical Care Medicine, Mie University School of Medicine, Tsu, JPN; 5 Department of Emergency Medicine, Emergency and Critical Care Center, Mie University Hospital, Tsu, JPN

**Keywords:** apache ii, mechanical ventilation, non-icu, predicted mortality, rural area

## Abstract

Introduction

Patients requiring mechanical ventilation (MV) are commonly managed in an intensive care unit (ICU); however, Japan is unique in that many patients are treated in non-ICU settings. The characteristics of these patients, nevertheless, are unknown. We sought to identify disease severity and MV settings of patients in non-ICU.

Methods

We retrospectively analyzed the clinical data of Kinan Hospital and Owase General Hospital, where there are no ICUs. Data for adult patients who required MV from January through December 2018 were collected. To find the characteristics of patients who have been decided to treat in non-ICU hospitals without early transferring, we analyzed patients who have been treated for more than three days in those hospitals.

Results

A total of 171 patients received MV; 29 patients were treated for more than three days. Of those, the mortality rate was 44.8% (13 patients). The median age was 80 years (range: 72-84 years). The mean Acute Physiologic Assessment and Chronic Health Evaluation II (APACHE II) score was 20.9 ± 8.1, and predicted mortality was 0.42 ± 0.25. Tidal volume per predicted body weight was 8.8 ± 2.1 mL/kg, and set inspiratory time was 1.6 ± 0.3 seconds.

Conclusions

We have first described the severity and the initial ventilator setting of MV patients treated for more than three days in non-ICU setting in Japan. The overall predicted mortality was 42%, and the average age of the patients was 80 years. Further research on wider areas and the comparison to the patients treated in ICUs are needed to identify the appropriateness of treating patients in non-ICU settings.

## Introduction

With the rising demand for mechanical ventilation (MV), the number of patients requiring admission to an intensive care unit (ICU) is increasing worldwide [1]. Recent and extensive spread of the coronavirus disease 2019 (COVID-19) has further increased the requirement for MV and ICU beds. In many countries, patients treated with MV are managed only in ICUs. However, in some regions or countries, including Japan, some of the patients treated with acute-phase MV are managed in non-ICU settings [2-5]. In Japan, the official ICU requirement is a ratio of 1:2 nurse-to-patient ratio, while 1:7 is enough for the general ward. Other regulation of “ICU” in Japan includes existence of specialized physician, enough area, etc. Thus, officially permitted ICUs only exist in larger hospitals, and many smaller hospitals have to transfer the patients or take care of them in the general ward where no specialized physicians or nurses are available. Previously, we reported that 46.4% of these patients (including those with noninvasive MV) are treated in non-ICU settings in Japan [2]. However, the patients’ disease severity and the ventilator settings were unknown. Therefore, it is not clear why non-ICU MV patients were not transferred to higher facilities; it could be that the patients’ symptoms were too mild or too severe. Thus, we would like to identify which types of patients have been decided to be treated in non-ICU settings without transferring. The purpose of this study was to describe the characteristics, including the severity score and ventilator settings, of patients treated with MV in non-ICU settings without transferring to higher facilities in the early phase.

The preprint version of this study has been published in Research Square [6].

## Materials and methods

We retrospectively analyzed the data of Kinan Hospital and Owase General Hospital, in Mie, Japan. There are no board-certified ICU physicians or board-certified emergency medicine physicians in those hospitals, and there are no ICU nurses in either hospital. The two hospitals are located in the East Kishu area; the ICU facility closest to the hospitals is about a 2-hour drive (approximately 150 km) by ambulance. Table 1 summarizes the characteristics of the two hospitals.

**Table 1:** Characteristics of two hospitals There are no ICU facilities in these hospitals.

	Owase General Hospital	Kinan Hospital
Location	Owase City in Mie Prefecture (about 100 km from Mie University Hospital)	Mihama-cho in Mie Prefecture (about 150 km from Mie University Hospital)
Bed	255 beds	244 beds
ICUs	None	None
Critical care board physician	None	None
Annual number of discharged patients (2018)	2,814	2,797
Annual number of discharged patients over 70 years old (2018)	2,087	2,084

**Figure 1:**
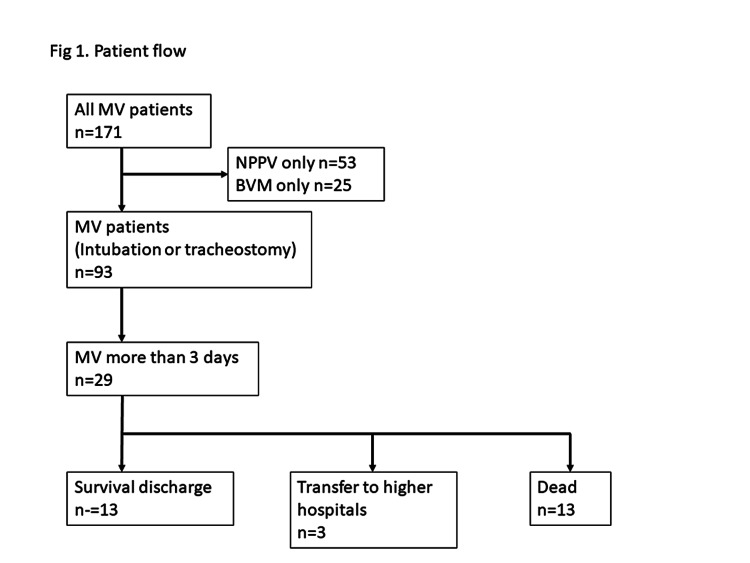
Patient flow A total of 171 patients received MV during the study period. NPPV or BVM only patients were excluded, and the number of patients who received MV for more than three days within the local facilities was 29. Overall, 13 survived to discharge, 13 died, and 3 were transferred to higher hospitals. BVM, bag–valve–mask ventilation; MV, mechanical ventilation; NPPV, noninvasive positive-pressure ventilation

The study period was from January through December 2018. The patient flow is shown in Figure 1. Inclusion criteria in the study were patients requiring MV either through tracheal intubation or tracheostomy for more than three days in those hospitals and age older than 18 years. We only included the patients who had been on MV for more than three days because we wanted to describe the characteristics of MV patients treated in non-ICU settings without transferring to higher facilities. Another reason to include those on MV for more than three days is to exclude short-term MV cases such as postoperative MV cases and patients who are on do not resuscitate order. The reason why we choose three days as a cut-off was that there might be some patients who have been decided to transfer to a higher facility but they could not do so because of the weekend. Exclusion criteria were terminal cancer, MV after cardiac arrest, and noninvasive ventilation. We excluded those patients also because we sought to describe the management of MV patients in smaller hospitals. Therefore, we thought one or two days of MV management were too short to evaluate the ability of intensive care in those hospitals. In fact, most of the MV patients treated for less than two days were cardiac arrest on arrival patients.

We collected the following patient data: age, mortality, arterial pressure, lactate concentration, number of MV days, Acute Physiologic Assessment and Chronic Health Evaluation II (APACHE II) score, outcome (discharge, death, or transfer), and MV settings for MV mode, positive end-expiratory pressure (PEEP), fraction of inspired oxygen (FiO_2_), tidal volume, and tidal volume/predicted body weight.

The predicted mortality was calculated using the equation described by Knaus et al. [7].

R = ek / (1 + ek)

k = −3.517 + (0.146 × APACHE II score)

+ 0.603 (only if postemergency surgery)

+ (diagnostic category weight)

This equation is based on the APACHE II score. APACHE II score is a commonly used severity score for the patients who were treated in the ICU. MV patients are usually treated in the ICU setting in all over the world, however not in Japan. Our patients might be treated in the ICU setting if there are enough ICUs in this area. Therefore, we thought that predicted mortality calculated using the APACHE II score indicates the predicted mortality if the patient was treated in ICU settings. Continuous variables were expressed as mean ± SD or as median (interquartile range), as appropriate. Continuous variables were compared using the t-test. A p-value of <0.05 was considered significant. Statistical analysis was performed using R statistical software (R Core Team, Vienna, Austria).

This study was approved by the Shimane University Institutional Committee on Ethics (Study number 4308; date: February 12, 2020).

## Results

According to the hospital announcement, during the study period, the annual number of patients admitted to these two hospitals was 5,611 patients, and the percentage of the people whose age was >70 years was 4,171 (74.3%).

Data were collected for a total of 171 patients requiring MV in the two hospitals for the one-year study period. Among them, 93 patients were treated by invasive ventilation, and 29 patients were treated for more than three days with MV. In total, 13 patients survived to discharge, 13 patients died, and three patients were transferred to a higher-level facility. The mortality rate was 44.8% (Figure 1).

The characteristics of the patients enrolled in the study are shown in Table 2.

**Table 2:** Patient characteristics APACHE, Acute Physiologic Assessment and Chronic Health Evaluation; Crea, creatinine; FiO_2_, fraction of inspired oxygen; Ht, hematocrit; MV, mechanical ventilation; Plt, platelet; WBC, white blood cell

Characteristics	N = 29
Age (years)	80 (72-84)
Male (%)	21 (72.4%)
Systolic blood pressure (mmHg)	90.7 ± 31.2
Mean blood pressure (mmHg)	61 ± 29.3
Respiratory rate (/min)	23 ± 8.2
FiO_2_	0.58 ± 0.24
PaO_2_ (mmHg)	122.7 ± 71.9
PaCO_2_ (mmHg)	63.1 ± 54.4
pH	7.32 ± 0.19
Na (mEq/L)	135.0 ± 7.1
K (mEq/L)	4.2 ± 0.7
Crea (mg/dL)	1.67 ± 2.5
Ht (%)	31.9 ± 8.4
WBC (/μL)	13600 ± 5400
Plt (/μL)	186000 ± 8700
Lactate (mg/dL)	40.7 ± 25.1
MV duration (days)	8 (4-19)
APACHE II	20.9 ± 8.1
Predicted mortality	0.42 ± 0.25

**Figure 2:**
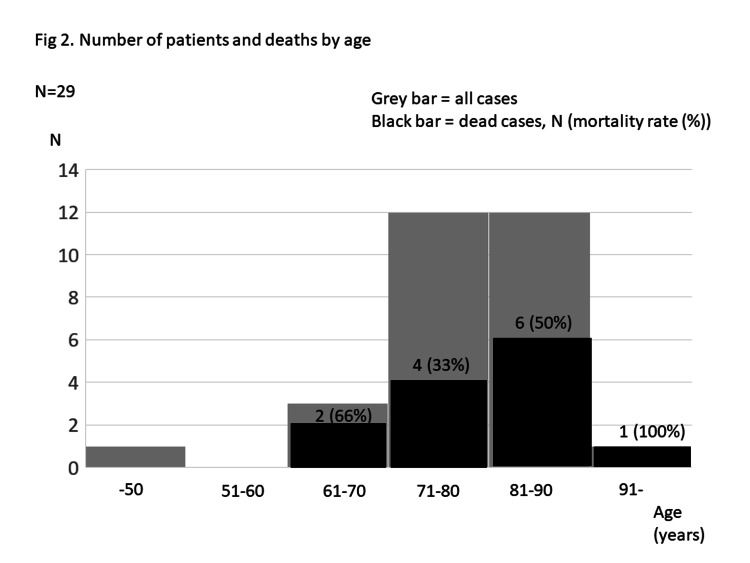
Number of patients and deaths by age This figure shows the number of patients and death by age. The grey bar shows the number of patients, and the black bar shows the number of dead cases.

The median age of the patients was 80 years, and the male:female ratio was 21:8. The number of cases and deaths are shown in Figure 2.

Mortality in patients grouped by age was 1/1 (100%) at ≥91 years of age, 6/12 (50%) at 81-90 years, 4/12 (33%) at 71-80 years, 2/3 (66%) at 61-70 years, and 0/1 (0%) at <50 years. The mean arterial pressure was 61 mmHg, and the lactate level was 31.5 mg/dL. The median duration of MV treatment was 8 days (range: 4-19 days), mean APACHE II score was 20.9 ± 8.1, and predicated mortality was 0.42 ± 0.25. The total number of ventilatory days for all patients added up to 725 days.

A comparison of patients who survived or died, excluding three patients who were transferred to higher facilities, is shown in Table 3.

**Table 3:** Comparison of patients who survived or died APACHE, Acute Physiologic Assessment and Chronic Health Evaluation; FiO_2_, fraction of inspired oxygen; MV, mechanical ventilation; PBW, predicted body weight; PEEP, positive end-expiratory pressure; TV, tidal volume

Characteristics	Survived, N = 13	Dead, N = 13	p-Value (95% CI)
Age	80.2	78.9	0.67 (-4.6 to 7.1)
APACHE II	17.1	24.9	<0.01 (-13.3 to -2.42)
Predicted mortality	0.32	0.57	<0.01 (-0.39 to -0.07)
MV duration (days)	10.7	28.0	0.27 (-48.9 to 14.3)
Mean blood pressure (mmHg)	60.0	64.5	0.65 (-25.3 to 16.0)
PaO_2_ (mmHg)	109	137	0.49 (-111 to 55.5)
PEEP (cmH_2_O)	6.36	6.15	0.79 (-1.43 to 1.85)
FiO_2_	0.49	0.70	0.02 (-0.39 to -0.02)
TV/PBW	8.39	9.32	0.36 (-3.02 to 1.17)

The APACHE II score (17.1 vs 24.9, p < 0.01) and corresponding predicted mortality rate (0.32 vs 0.57, p < 0.01) were significantly lower in patients who survived. Age (80.2 vs 78.9 years, p = 0.67), mean blood pressure (60.0 vs 64.5 mmHg, p = 0.65), MV duration (10.7 vs 28.0 d, p = 0.27), and tidal volume/predicted body weight (8.39 vs 9.32 mL/kg, p = 0.36) were not different between the groups. Survival cases showed significantly lower FiO2 requirement than dead cases (0.49 vs 0.70, p = 0.02).

The MV settings are described in Table 4.

**Table 4:** Mechanical ventilator settings A/C, assist/control; FiO_2_, fraction of inspired oxygen; PBW, predicted body weight; PEEP, positive end-expiratory pressure; SIMV, synchronized intermittent mandatory ventilation; Spont, spontaneous

	N = 29
Mode of ventilator (n, %)	
A/C	6 (21%)
SIMV	16 (55%)
Spont	4 (14%)
Data not recorded	3 (10%)
FiO_2_	0.58 ± 0.24
PEEP (cmH_2_O)	6.1 ± 1.7
Inspiratory time (setting) (seconds)	1.6 ± 0.3
Peak pressure (cmH_2_O)	19 ± 4.4
Tidal volume (ml)	442 ± 106
Tidal volume/PBW (mL/kg)	8.8 ± 2.1

The ventilator modes were as follows: assist/control mode (21%), synchronized intermittent mandatory ventilation (SIMV) mode (55%), and spontaneous mode (14%); data were not recorded in 10% of patients. The average PEEP setting was 6.1 ± 1.7 cmH_2_O, and inspiratory time setting was 1.6 ± 0.3 seconds. Tidal volume per predicted body weight was 8.8 ± 2.1 mL/kg. Tidal volume was not recorded in 7/29 (24%) patients, inspiratory time was not recorded in 21/29 (72%) patients, and respiratory rate was not recorded in 9/29 (31%) patients.

## Discussion

We have described the clinical characteristics of patients who were treated with MV in a non-ICU setting in East Kishu area, Japan. In this area, 29 patients were treated with MV at local hospitals that did not have ICU facilities or ICU physicians. The mortality rate was 44.8%, the median age of the patients was 80 years, and the APACHE II score was 20.9 ± 8.1. The average mean blood pressure was 61±29.3 mmHg, median MV duration was 8 days (range: 4-19 days), and average FiO_2_ at initial setting was 0.58 ± 0.24. As we have shown in a previous study, many MV patients are treated in non-ICU settings [2]. However, the severity of those patients has never been described. These are the first descriptive data.

Since these are the first descriptive data of non-ICU MV patients, it is difficult to evaluate or compare to other data. Patients with an APACHE II score of more than 20 are generally thought as severe and appropriate to enter the ICUs. The characteristics of averages of the patient’s data are hypotensive with elevated lactate concentration and requiring higher FiO_2_. In Japan, the Ministry of Health, Labour, and Welfare define the criteria of entering the ICUs: patients with coma, acute respiratory failure, acute heart failure, acute drug intoxication, shock, serious metabolic disorder, extensive burns, after major surgery, after resuscitation, and other serious conditions. Thus, our data indicate that the average severity of the patients was enough to meet the criteria of entering the ICU. On the other hand, nearly half of the patients were over 80 years old. Therefore, physicians in those hospitals might be reluctant to transfer the patients to ICU settings because of the patients’ old age.

Univariate comparison of survival and dead cases showed that severity score, predicted mortality rate, and initial FiO_2_ were statistically different. These data might indicate that patients who require higher oxygen should be transferred to higher facilities. However, there is another possibility that these patients were too severe to treat even in higher facilities. Further analysis is needed. The median MV duration was 8 days (range: 4-19 days). The total number of ventilatory days for all patients added up to 725 days. This may indicate at least one MV patient is treated in those hospitals almost every day. This result was comparable with our previous study, in which we completed an attitude survey for physicians in hospitals without ICU facilities, and around 10% of physicians answered that they treat more than 11 patients with MV in a year [8]. Despite this study being a pilot study analyzing only one region, the data were comparable to the national survey.

To further describe the characteristics of the MV patients, the MV settings are also researched. As a result, the average tidal volume per predicted body weight was 8.8 ± 2.1, SIMV mode was the most preferred mode of ventilation, and the inspiratory time was not recorded in 72% of the cases. The evidence shows that the tidal volume per body weight should be less than 6 mL/kg [9]; therefore, the average of the tidal volume was little higher. However, from the worldwide epidemiological survey on MV setting in the ICU, 34% of the patients with acute respiratory distress syndrome received more than 8 mL/kg of predicted body weight [10]. Thus, this practice might be comparable to ICU management. The SIMV mode was previously the preferred mode in ICUs, but recent studies revealed that it increased the duration of weaning [11] and may increase patient-ventilator asynchrony [12,13]. It is therefore not a preferred mode in ICUs. Surprisingly, respiratory rate was not recorded in 31% (9/29) of the patients and inspiratory time was not recorded in 72% (21/29) of the patients. Higher inspiratory time also induced patient-ventilator asynchrony and resulted in ventilator-induced lung injury [14]. Because patient-ventilator asynchrony is a relatively new concept, it might be difficult for non-ICU physicians who are not board-certified in critical care to catch up with emerging evidence in the critical care field. These results indicate that regular updates in standard MV education might not enough for non-ICU professionals. Further research on how MV settings and their change influence the outcomes is needed.

There are three main limitations of this study. First, the study was conducted only in one area of Japan. This result may not represent the nation as a whole. Further analysis including many areas of Japan is needed. Second, because our main purpose was the description of the characteristics of MV patients treated in non-ICU settings, we could not compare the outcomes in non-ICU patients and ICU patients. Thus, it is difficult to identify the appropriateness of managing MV patients in non-ICU settings. Comparison of MV patients treated in non-ICUs and ICUs are needed to evaluate this. Third, we only described the initial MV settings. The MV settings might be changed appropriately after further treatment. Longer analyses are needed to evaluate the appropriateness of MV settings.

## Conclusions

We have first described the severity and the initial ventilator setting of MV patients treated for more than three days in non-ICU settings in Japan. The overall predicted mortality was 42%, and the average age of the patients was 80 years. The lack of recording inspiratory time and respiratory rate were observed. Further analysis, including a wider area and comparison of non-ICU and ICU settings are required to evaluate the appropriateness of Japanese ICU policy.
